# Cost-effectiveness of pain management services for chronic low back pain: a systematic review of published studies

**DOI:** 10.1186/s12913-020-5013-1

**Published:** 2020-03-12

**Authors:** Saja H. AlMazrou, Rachel A. Elliott, Roger D. Knaggs, Shiekha S. AlAujan

**Affiliations:** 1 Clinical pharmacy department, College of Pharmacy, King Saud University, Riyadh, Saudi Arabia; 2 Manchester Centre for Health Economics, School of Health Sciences, The University of Manchester, 4th floor, Jean Mcfarlane Building, Oxford Road, Manchester, M13 9PL UK; 3 Primary Integrated Community Solutions Ltd, Nottingham Business Park, Nottingham, NG8 6PY UK; 4 Division of Pharmacy Practice & Policy, The School of Pharmacy, University of Nottingham, University Park, Nottingham, NG7 2RD UK

**Keywords:** Cost effectiveness, Pain management services, Back pain

## Abstract

**Background:**

Chronic low back pain (CLBP) is a highly prevalent condition that has substantial impact on patients, the healthcare system and society. Pain management services (PMS), which aim to address the complex nature of back pain, are recommended in clinical practice guidelines to manage CLBP. Although the effectiveness of such services has been widely investigated in relation to CLBP, the quality of evidence underpinning the use of these services remains moderate. Therefore the aim is to summarize and critically appraise the current evidence for the cost effectiveness of pain management services for managing chronic back pain.

**Methods:**

Electronic searches were conducted in MEDLINE, EMBASE and PsycINFO from their inception to February 2019. Full economic evaluations undertaken from any perspective conducted alongside randomized clinical trials (RCTs) or based on decision analysis models were included. Cochrane Back Review Group (CBRG) risk assessment and the Consolidated Health Economic Evaluation Reporting Standards (CHEERS) checklist were used to assess the methodological quality of eligible studies.

**Results:**

Five studies fulfilled eligibility criteria. The interventions varied significantly between studies in terms of the number and types of treatment modalities, intensity and the duration of the program. Interventions were compared with either standard care, which varied according to the country and the setting; or to surgical interventions. Three studies showed that pain management services are cost effective, while two studies showed that these services are not cost effective.

In this review, three out of five studies had a high risk of bias based on the design of the randomised controlled trials (RCTs). In addition, there were limitations in the statistical and sensitivity analyses in the economic evaluations. Therefore, the results from these studies need to be interpreted with caution.

**Conclusion:**

Pain management services may be cost effective for the management of low back pain. However, this systematic review highlights the variability of evidence supporting pain management services for patients with back pain. This is due to the quality of the published studies and the variability of the setting, interventions, comparators and outcomes.

## Background

Low back pain is a common health condition; the estimated global mean point prevalence is 11.9% [1]. In the United Kingdom (UK), musculoskeletal pain is the leading cause of chronic pain, with low back pain and osteoarthritis together responsible for over half of all cases [2]. According to the Health Survey for England [3], back pain was the second most commonly reported site of pain. Low back pain affects several life domains [4]. Patients with back pain report limitation in daily activities such as work, housework and leisure activities. Low back pain affects relationships, increases feelings of loneliness, cohabitation problems, and concerns surrounding sexual relations, and reduces social interaction. Low back pain has been suggested to be the leading cause of disability worldwide [5], with an associated substantial economic burden. The indirect cost due to productivity loss represent a large proportion of the overall cost; a systematic review of 27 cost-of illness studies conducted worldwide showed that back pain has a major impact on indirect costs, which can represent 50 to 89% of the total costs [6].

The biopsychosocial model is considered the most valid framework in understanding and managing chronic low back pain (CLBP) [7]. The model addresses pain as a complex and dynamic interaction between physiological, social and psychological elements. In order to manage pain within a biopsychosocial framework a multidisciplinary management approach to address these elements is required [8]. This model requires collaboration between healthcare professionals from different specialties in order to deliver a multidimensional and comprehensive treatment plan.

Combined physical and psychological treatment programs which fulfil the criteria of multidisciplinary pain services have been recommended in clinical practice guidelines, including in the UK, as a rational option for managing people with CLBP [9, 10]. A recent systematic review of the evidence for the clinical effectiveness of multidisciplinary pain management services on clinically relevant outcomes in CLBP examined 41 randomized controlled trials (RCTs) targeting adults with CLBP for more than 12 weeks [11]. The results demonstrated a moderate effect in favour of the pain management services (PMS) in improving disability and pain.

The cost-effectiveness of pharmacological treatments for CLBP has been assessed by Haas et al. [12]. The systematic review included seven economic evaluations, both model-based and those carried out alongside RCTs. The results highlighted variable quality of clinical effectiveness data (RCTs), due to sample size not being sufficient to detect differences in outcomes, or convenience sampling being used in non-randomised studies. The quality of some of the economic evaluations was affected by not clarifying the study perspective or omitting to discount future costs or benefits. The results from this review have limited relevance to PMS due to fundamental differences between pharmacological and pain services due to combination of several treatment modalities and their intensity.

Given the increasing prevalence of CLBP [1] and the moderate effect size and perceived high delivery costs of PMS [13], there is a need to critically appraise the cost-effectiveness of these services to support stakeholders and decision-makers in the commissioning and reimbursement of PMS. The aim of this systematic review is to summarise and critically appraise the current evidence for the cost effectiveness of PMS in managing CLBP.

## Methods

### Types of studies

Full economic evaluations (i.e. cost effectiveness, cost minimisation, cost-benefit, cost utility and cost consequences analysis), undertaken from any perspective and conducted alongside RCTs or based on decision analysis models, were included.

Partial economic evaluations, published protocols, conference papers and observational studies, such as cohort, case control and non-randomised studies, were excluded.

### Types of participants

Studies including adults (age > 17 years) who had been referred to PMS from primary care with CLBP were eligible. The Cochrane Back Review Group (CBRG) does not recommend mixing studies about acute and chronic low back pain as the underlying causes differ and, therefore, response to the intervention might be systematically different [14] . Therefore, people with acute or sub-acute low back pain lasting for less than 3 months were excluded. People with low back pain caused by cancer, infection, cauda equina syndrome or inflammatory disorders, such as spondylitis, were excluded, as these types of people require intensive and urgent assessment in secondary care.

### Types of interventions

Although PMS have been recommended in clinical practice guidelines, there is still no consensus around the exact definition of these services [15]. Based on the systematic review by Kamper et al. [11], PMS are defined as health services targeting at least two of the social, physical, psychological and/or occupational aspects provided by one or more healthcare professionals. Given the wide range of terms used to describe these services, we included all relevant terms in our search strategy. The terms including multidisciplinary, interdisciplinary, multimodal, multiprofessional, pain clinics and rehabilitation clinic. An inclusive strategy was adopted in order to identify all relevant publications and then a detailed assessment of the service was undertaken.

PMS based in primary and secondary care were eligible. The components of the services could include pharmacological treatments, physiotherapy, cognitive behavioural therapy, complementary medical approaches, such as acupuncture, and other relevant specialties, including rehabilitation medicine, occupational therapy and social services. PMS that offered fewer than two treatment modalities within the service were excluded.

### Types of outcome measures

PMS can aim to either improve functional disability and pain or focus on the vocational outcomes, such as return to work. Therefore, the complex multidimensional aims of these services require the measurement of a variety of outcomes that best address the study aims and research questions. Patient-reported outcomes, such as pain intensity, functional disability, quality of life and return to work, were assessed. Healthcare resource utilisation was also examined.

### Search methods for study identification

Electronic searches were conducted in MEDLINE, EMBASE and PsycINFO from their inception to February 2019. The Health Technology Assessment Database (HTA) in the Centre for Reviews and Dissemination (CRD) were also searched. The NHS Economic Evaluation Database (NHS EED) was searched from inception to February 2016. Reference lists were checked to identify relevant publications.

The search was restricted to studies about humans and published in English. The terms used in the search strategy are listed in Additional file 1 .

### Methodological quality assessment

The Consolidated Health Economic Evaluation Reporting Standards (CHEERS) checklist was used to assess the methodological quality of reporting economic studies [16]. Where the economic evaluation was linked to an RCT, the 12-item checklist recommended by the CBRG was used to assess the RCT’s risk of bias [14, 17]. This tool addresses several domains related to a study’s internal validity. According to the CBRG guidelines [14, 17], a study is considered to have a “low risk of bias” if at least six domains were categorised as having a low risk of bias. Two reviewers (SA1 & SA2) screened the titles and abstracts and then one reviewer extracted the data from the eligible studies. A third reviewer (RAE) resolved disagreements if necessary.

The extracted data from each study contained author name, country, year, participant characteristics, intervention, comparator and outcomes. Data allowing assessment of risk of bias and adherence to CHEERS quality criteria were extracted.

## Results

**Fig. 1:**
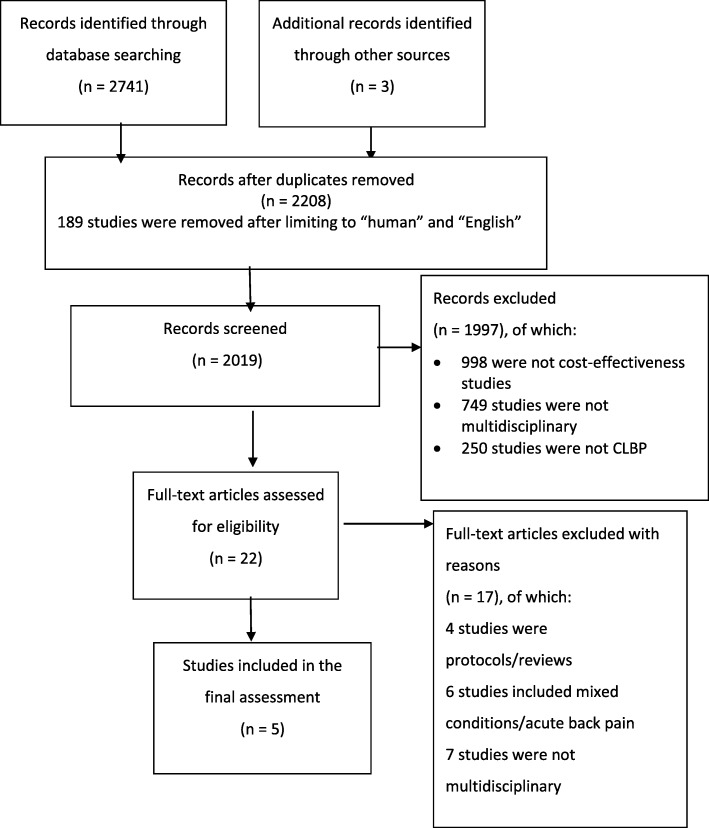
Flow Chart Diagram

The initial search retrieved 2744 publications. After removing duplicates, and limiting to ‘English’ and ‘human’, the results numbered 2019. Of the 2019 results, 1997 studies were judged, via the title and abstract, as not relevant to the scope of this review. The flow chart diagram of the included studies and the reason that studies were excluded is presented in Fig. 1.

The remaining 22 full-text articles were assessed for eligibility. Only five studies fulfilled our inclusion criteria. Seventeen studies were excluded and details of the excluded studies and the reasons for exclusion are described in Additional file 2 .

### Study characteristics

Five studies were included in the review; two studies were conducted in Norway [18, 19], two in the Netherlands [20, 21] and one in the UK [22]. These studies were published between 2002 and 2014. The characteristics of the included studies are summarised in Table 1. The total number of participants in the five studies was 1023, with the sample size ranging from 134 to 349 people. Two of the studies only included adults in employment taking sick leave due to CLBP [19, 20]. The mean age of the participants ranged from 41 to 45.5 years, with equal gender distributions. The mean duration of CLBP symptoms ranged between 5 and 8 years.

**Table 1:** Characteristics of the included studies

Study ID	Setting	Target population/sample size	Intervention	Comparator(s)
Skouen 2002 [19] Norway	Outpatient spine clinic	Patient sick listed for at least 8 weeks or employees not on sick leave but sick listed for at least 2 months /year *n* = 195	Light multidisciplinary programme (assessment by physiotherapist and psychologist + individual education and feedback) Extensive multidisciplinary programme (4-week programme - 6 h session 5 days/week) consisting of CBT, education, exercises, work place intervention)	Usual care
Rivero-Arias 2005 [22] United Kingdom	Secondary care	18–55 years with CLBP > 1 year *n* = 349	Intensive rehabilitation programme (education + exercise) led by a physiotherapist and clinical psychologist	Spinal stabilisation surgery
Smeets 2009 [21] Netherlands	Primary care	Age between 18 and 65 years with CLBP> 3 months with RMD score > 3 *n* = 172	Active physical treatment + graded activity + problem training	2 comparators: active physical treatment and graded activity plus problem training
Lambeek 2010 [20] Netherlands	Primary and secondary care	Adults (18–65) with CLBP in paid work for at least 8 h/week on partial sick leave *n* = 134	Integrated care programme (workplace intervention + graded activity)	Usual care
Johnsen 2014 [18] Norway	Hospital	Adults with CLBP > 1 year with degenerative change in lumbosacral intervertebral disc *n* = 173	Multidisciplinary rehabilitation (outpatient programme focussed on exercise and CBT delivered by a physiotherapist and physical medicine specialist)	Total disc replacement surgery

*CLBP* chronic low back pain, *CBT* cognitive behavioural therapy, *RMD* Roland Morris Disability

A wide range of outcome measures were used in the studies included in this review. Return-to-work (RTW) was the primary outcome in three studies [19, 20, 22]. Four studies used two measures for disability [18, 20–22]. Regarding the baseline measures of disease severity, the mean functional disability score using the Roland Morris Disability Score (RMDS) [23] was 14, the score can range between 0 and 24 [20, 21]. Whereas the Oswestry Disability Index (ODI) [24] mean score was 43 [18, 22], the ODI score ranges between 0 and 100. The mean pain intensity score from the three studies was 5.8, with the possible ranges of pain intensity being between 0 and 10 [18, 20, 21]. For all measures, the high scores identify the greatest disability and pain. The mean EQ-5D-3 L [25] score ranged from 0.26–0.49 [18, 21, 22]. A generic health status measure, generally, the possible ranges for the EQ-5D-3 L are between 0 and 1, where high scores mean better health status. A further generic health status measure, the Short Form 6D [26], was also used in conjunction with EQ-5D-3 L in one study [18].

One study looked at costs from the patient and healthcare provider perspectives [22], while the remaining studies were conducted from the societal perspective [18–21]. The length of follow-up was between 12 and 24 months.

The PMS consisted of combinations of cognitive behavioural therapy, physical therapy and workplace interventions. Two studies compared PMS with usual care [19, 20], two with surgery [18, 22] and one with physical treatment and graded activity (a treatment that includes behavioural and cognitive methods to improve activity endurance) (two comparators) [21]. The outcomes were return to work [19, 20], quality-adjusted life-years (QALYs) using EQ-5D-3 L [18, 21, 22] and disability using RMDQ [21] and ODI [18, 22].

### Design and description of pain management services

The studies were delivered in a secondary care setting only [18, 19, 22], primary care only [21], or a combined setting [20]. All studies clearly described the service in terms of treatment modalities and the staff involved in delivering these services. However, in some studies the duration of treatment varied between people within the study [18, 20]. Among the included the duration ranged between one and 3 months and the total number of hours ranged between 3 and 75 h. In another study, the intensity of treatment in terms of the total hours provided was not consistent between individuals in the study [22]. Study participants were generally working-age adults; two studies focused on employees with CLBP [19, 20] and no studies included people above 65 years old.

The description of services provided in the included studies is summarised in Table 2.

**Table 2:** Service description in the included studies

Study ID	Staff delivering the intervention	Intervention description	Number of hours/day and duration	Notes
Skouen 2002 [19] Norway	PT Nurse Psychologist (if necessary)	Light intervention: Assessment, a lecture, three individual sessions and individually-based graded exercise.	Assessment (1–2 h) Lecture (1 h) Total for individual sessions (45 min)	Some people were offered visits to an external PT, local National Health Insurance and workplace visits.
		Extensive intervention: Educational sessions, exercises and (occasional) workplace intervention	6 h, 5 days a week for 4 weeks.	
Rivero-Arias 2005 [22] United Kingdom	PT and clinical psychologist	Education Exercises and hydrotherapy	5 days a week for 3 weeks The total hours were 60–110 (75 h on average)	One of the centres did not have a psychologist or hydrotherapy sessions.
Smeets 2009 [21] Netherlands	PT Clinical psychologist social worker	Physiotherapy Graded activity with problem-solving training	Physiotherapy: 105 min three times a week for 10 weeks Graded activity: 19 sessions starting from 3rd week.	
Lambeek 2010 [20] Netherlands	OT physician PT OT medical specialist	Workplace intervention Graded activity	Graded activity (26 sessions) for 3 months or until RTW	Coordinated
Johnsen 2014 [18] Norway	PT Specialist in physical medicine	Lectures and individual discussions Daily workout	60 h over 3–5 weeks	Nurse, psychologist or social worker as needed

*PT* Physiotherapist, *OT* Occupational therapist

### The comparator

Two studies, which were conducted in the Netherlands and Norway, compared PMS with “standard care” [19, 20]. In Lambeek et al., standard care consisted of family physician visits, in addition to occupational therapist consultations, provided in a primary care setting [20]. In Skouen et al., standard care consisted of examination in the spine outpatient clinic by a physician, followed by referral back to the GP [19].

Two studies compared the effect of PMS with surgery [18, 22]. The surgical procedures were total disc replacement [18] and spinal stabilisation [22]. As surgical options are usually reserved for the severest cases, the patient populations in these studies are likely to be different from those where GP/medical management is offered as standard care [19–21]. This is demonstrated by the increased pain intensity and lower quality of life at baseline in the surgical studies [18, 22].. The mean baseline utility scores (EQ-5D) were 0.26 and 0.49 in studies assessing surgery [18] and non-surgical treatments [21] as comparators, respectively. Similarly, the baseline pain intensity score was 6.9 in the study assessing surgery [18], while in the study assessing standard care, the baseline score was 5.7 [20]. Therefore outcomes achieved from referral to PMS are not comparable to other studies due to higher baseline pain and disbility levels.

### Methodological design

All of the economic evaluations were conducted alongside RCTs. The risk of bias assessment of the included studies is described in Table 3.

**Table 3:** Risk of bias assessment according to the Cochrane Back Review Group (CBRG)

Risk of bias item	Skouen 2002 [19]	Rivero-Arias 2005 [22]	Smeets 2009 [21]	Lambeek 2010 [20]	Johnsen 2014 [18]
Was the method of randomisation adequate?	Low	Low	Low	Low	Low
Was the treatment allocation concealed?	Low	Unclear risk	Low	Low	Low
Was the patient blinded to the intervention?	Not possible				
Was the care provider blinded to the intervention?					
Was the outcome assessor blinded to the intervention?					
Was the dropout rate described and acceptable?	Low	High	Low	Low	Low
Did the analysis include intention-to-treat analysis?	Low	Low	Low	Low	Low
Are the study reports free of suggestion of selective outcome reporting?	Unclear risk	Unclear risk	Unclear risk	High	Unclear risk
Were the groups similar at baseline?	Unclear risk	Low	Low	Low	High
Were co-interventions avoided?	Unclear risk	High	High	Unclear risk	Unclear risk
Was the compliance acceptable in all groups?	Unclear risk	High	High	High	Unclear risk
Was the timing of outcome assessment similar in two groups?	Low	Low	Low	Low	Low
Summary risk of bias	HIGH	HIGH	LOW	LOW	HIGH

Unclear = item not reported clearly

The study will be considered to have a low risk of bias if 6 or more items are satisfied, otherwise it will be considered to have a high risk of bias

Two of the five studies were considered to have low risk of bias [20, 21]. The major strengths in all of the included studies were that the methods of randomisation and allocation were clear. Moreover, intention-to-treat analysis was considered in the statistical analysis for missing data.

High risk of bias was identified in three studies [18, 19, 22]. Two studies reported that the intervention group received extra visits to physiotherapists and other healthcare professionals compared with the standard care arm [21, 22]. Hence, the intervention group might have had better outcomes, compared with standard care, because of these additional visits. One of the important aspects in assessing the quality of RCTs is the sample size and statistical power [27]. Four out of five studies were sufficiently powered (power threshold 80%) to detect a difference in functional disability using the ODI [18, 22], RMDS [21] or return to work [20].

Although all included studies incorporated RCTs, randomisation by itself does not ensure that the baseline characteristics of the study participants in the comparator groups are similar [27]. Knowing this information is essential to demonstrate that the participant response to treatment is directly attributed to the intervention effect and not to other patient-related factors. Adjusting effect size for baseline characteristics should be performed using statistical methods, generally regression. In our review, only one study [21] performed regression to adjust effect size for baseline characteristics. Furthermore, two studies clearly reported that there was a significant difference between the study participants at baseline, which they did not then go on to adjust [18, 19].

The quality of reporting economic evaluations in terms of costs and outcomes is reported in Table 4, while the details of sensitivity analysis and the results are summarised in Table 5.

**Table 4:** Assessment of economic evaluations based on CHEERS criteria (inputs to economic evaluation: costs and outcomes)

Study ID	Currency/year	Direct cost	Indirect costs	Time horizon	Health outcome	Valuation of preference outcomes
Skouen 2002 [19]	Norwegian Krone, price of clinic in 1996, no inflation	Top down approach	Yes	26 months	Return to work	NA (utilities were not collected)
Rivero-Arias 2005 [22]	2002–2003 GBP inflated to base year (2005)	Bottom up approach	Yes. costing total hours worked by each patient	24 months	Return to paid employment, total hours worked, utility using EQ. 5D	Social tariff from representative sample of UK population
Smeets 2009 [21]	2003 Euros	Top-down approach and costing diaries	Yes, using human capital approach	12 months	Disability using RMDQ,utility using EQ. 5D	AUC, population and techniques were not described
Lambeek 2010 [20]	Index year 2007 (Euro converted to GBP)	Bottom up approach	Yes, using human capital approach	12 months	Return to work, utilities using EQ. 5D	Dutch tariff however no description of population or methods used
Johnsen 2014 [18]	Norwegian Krone with 2006 as a base year. Costs were adjusted for inflation into 2012 prices and converted to Euros using the rate 1 € = 6.7 Kr2006	Top-down approach and costing diaries	Yes, using human capital approach	24 months	Utilities using EQ. 5D and SF-6D	QALY was estimated as AUC using trapezoidal method. Population and techniques were not addressed.

*AUC* area under the curve, *CE* cost effectiveness, *CB* cost benefit, *CU* cost utility, *QALY* quality adjusted life years, *EQ. 5D* EuroQol 5 dimensions, *SF-6D* Short Form 6 dimension, *GBP* British pound, *NA* not applicable

**Table 5:** Economic evaluation based on CHEERS criteria (statistical analysis and results)

Study ID	Statistical analysis of cost data	Statistical analysis for missing data	Incremental economic analysis reported	Sensitivity analysis	Difference in outcome	Difference in costs	Difference in outcome and costs
Skouen 2002 [19] Norway	Not reported, only mean difference in outcome evaluated by ANOVA. RR and 95% CI for the effect of intervention versus comparator on outcome.	Not reported	productivity gain = NoK 7,858,100 - cost, the net productivity gain = 240,900	Not performed	Yes	Not reported	No
Rivero-Arias [22] 2005 UK	Arithmetic mean for cost and outcomes	Intention to treat and multiple imputation (variance correction)	Bootstrapping (1000 replications)	Yes	No	Yes	Yes
Smeets 2009 [21] Netherland	student t test for change in outcomes, linear regression to adjust for baseline differences	Intention to treat analysis, missing data were replaced by mean score of cost and utility	Bootstrapping (1000 replications)	Yes	No	No	No
Lambeek [20] 2010 Netherlands	Not reported	Intention to treat analysis, multiple imputation	Bootstrapping (5000 replications)	Yes	Yes	Yes	Yes
Johnsen 2014 [18] Norway	Student t test for cost and utilities	Intention to treat analysis, multiple imputation	Bootstrapping (10,000 replications)	Yes	Yes	Yes	Yes

*ANOVA* analysis of variance, *RR* relative risk, *CI* confidence interval, *MDT* multidisciplinary treatment, *NoK* Norwegian Krone

### Healthcare resource use and cost

In this review, all of the studies included direct medical and indirect costs. Four studies included direct non-medical costs, such as travel expenses [18, 20–22]. Four studies took the societal perspective [18–21] and one study took the healthcare provider perspective [22]. Although the last study stated that they conducted their evaluation from a healthcare provider perspective, indirect costs were calculated. Although Skouen et al. stated that their study took a societal perspective [19], direct non-medical costs were not collected.

There are two methods of assessing the service costs, the top-down and the bottom-up (micro-costing) approaches [28]. The top-down approach divides the total budget of a health intervention by the total number of people to give an “average” estimate of cost per patient, whereas the bottom-up approach uses patient-level resource use data to generate costs. The latter is the preferred method in economic evaluations to account for variations in costs between study participants [28]. In this review, three studies used the top-down approach [18, 19, 21], while two studies used the bottom-up method [20, 22]. The method of collecting costs was implied, rather than clearly stated, in three studies [19, 21, 22]. Only two studies clearly reported all resource use and their unit costs [20, 21]. In two studies, some unit costs were missing [18, 22] and Skouen et al. did not report unit costs [19].

In this review, two studies used postal questionnaires to collect resource use data from people [20, 22], which might be subject to recall bias, especially if the recall period is more than 3 months [29]. In Lambeek et al., the recall period was 3 months [20] while, in Rivero-Arias et al., the recall period was 6 months and 1 year [22]. Two studies used costing diaries to collect resource use data [18, 21]. Costing diaries aim to collect data prospectively, which reduces the risk of recall bias. To minimise the risk of incompletion, regular telephone reminders are recommended but neither of the studies using diaries reported providing reminders [18, 21].

Productivity loss due to illness can be accounted for by absenteeism, the inability to attend work, and presenteeism, the reduced functionality in terms of quality and quantity while working [30, 31]. Productivity loss can be measured either objectively, by using attendance records, or subjectively, using self-report by the employee [30]. These methods have some limitations; objective measures might be inaccurate for assessing presenteeism, as they only record employee attendance, with no emphasis on productivity levels in terms of quality.

All studies assessed the effect of PMS on productivity loss. Absenteeism was the only work outcome evaluated. Four studies clearly reported their methods of collecting productivity loss [19–22]. Although the appropriate recall period is still inconclusive, 3 months’ recall for absenteeism and 1 week for presenteeism is recommended [30]. Two studies used “monthly” self-reported methods, utilising calendars [20] and diaries [21]. In another study [22], the employment status was self-reported over a relatively long period of 6 months and 1 year with insufficient information about the measurement method to assess quality. An objective measure was used in one study [19], which utilised the national health insurance registry to assess sickness absence. Johnsen et al. [18] did not report the method of data collection.

In order to value productivity loss among employees, the “human capital approach” and the “friction cost method” can be used [30]. As the friction cost approach can produce lower estimates of cost, it is recommended to use both approaches to determine any methods-dervied difference. Four studies used the human capital approach alone to value productivity loss [18–21]. In Rivero-Arias et al. study the productivity was assessed by calculating the total hours worked by each patient at baseline, six,tweleve and 24 months [22].

### Statistical analysis

The statistical analysis of patient-level cost data needs to be adjusted from standard approaches as cost data are generally “positively skewed”, because a small number of people usually require more healthcare resources [28, 32]. Non-parametric tests rely on medians and distributional shape. Non-parametric bootstrapping with replacement is the preferred method to analyse cost data because it compares arithmetic means while avoiding distributional assumptions. Standard parametric tests can be used to analyse cost data only if the sample size is large, where skewness will not affect the validity of the analysis. Barber et al. reported that, for sample sizes larger than 150 participants [33], the t-test is usually robust and valid, as parametric assumptions will generally hold. In this review, two studies used non-parametric bootstrapping to test the difference in cost [20, 21], whereas two studies with larger sample sizes, 349 [22] and 173 [18], used parametric t-tests for cost analysis. Skouen et al. did not analyse differences in costs [19].

Discounting is used to estimate the future value of outcomes and costs and assumes present outcomes and costs are considered more valuable than those in the future [28]. Future costs and outcomes should be discounted where follow-up is longer than 1 year, using nationally preferred discount rates. Lack of discounting can lead to inaccuracy in estimating the cost-effectiveness results. In this review, three studies had interventions that continued for 2 years [18, 19, 22], two of which reported the discount rate according to the country-specific rates [19, 22].

### Dealing with uncertainty

The incremental cost effectiveness ratio (ICER) is the main summary measure of an economic evaluation and is the difference in cost divided by the difference in effect (outcome) between two interventions [28]. The base case analysis generates the ICER from the preferred outcome and cost data. Sensitivity analysis is used to test the sensitivity of the ICER to variation in cost and outcome parameters used in the base case analysis [28, 32]. In one-way sensitivity analysis, one parameter is changed at a time to test the results. Multiple-way analysis changes multiple parameters at the same time. Although one-way sensitivity analysis is easy and understandable, it can underestimate total uncertainty in the ICERs [34].

Probabilistic sensitivity analysis (PSA) assumes that the values of input cost and outcome variables have a probability distribution. Probabilistic incremental economic analysis is usually carried out using bootstrapping to generate credibility intervals that provide a quantitative measure of uncertainty around ICER point estimates (“expected value”). For the graphical representation of ICERs, cost effectiveness planes are used to present the distribution of bootstrapped ICERs [28]. Another common graphical presentation used in economic evaluation is the cost effectiveness acceptability curve (CEAC) [28]. The CEAC is a technique for representing information on uncertainty in cost-effectiveness. A CEAC demonstrates the probability that an intervention is cost-effective compared with the substitute, given the observed data, for a range of maximum monetary thresholds that policy makers are willing to pay for a specific unit change in effect [35].

In this review, all studies carried out one-way sensitivity analysis. Four studies generated ICERs using bootstrapping [18, 20–22] and three of them presented ICERs on cost effectiveness planes [18, 20, 21]. CEACs were used in these studies to present the probability of cost effectiveness [18, 20–22].

### Cost-effectiveness of PMS

The ICERs generated by the studies are summarised in Table 6 . Only one study concluded that PMS dominates usual care (more effective and less costly) [20]. Skouen et al. concluded that multidisciplinary services are cost-effective in men only [19]. However, this conclusion needs to be interpreted with caution given that top-down costs were used and there was no sensitivity or statistical analyses reported. Two studies reported that PMS are cheaper and less effective than surgery [18, 22]. Therefore, a trade-off between cost and effect needs to be considered. Smeets et al. compared PMS with active physical treatment (APT) and graded activity plus problem solving (GAP) [21]. In this study, the PMS was dominated when compared with GAP, while it was cheaper and less effective when compared with APT.

**Table 6:** Summary of incremental cost effectiveness analysis results

Study ID	Intervention/Comparator	Outcome measure	Intervention cost	Cost difference	Outcome difference	ICER
Skouen 2002 [19] Norway	Multidisciplinary treatment vs usual care	Return to work	NoK 9023	Not reported	Net productivity gain NoK 7,858,100 (USD 924500)	Net productivity gain = NoK 7,240,900(USD 852000)
Rivero-Arias 2005 [22] UK	Intensive rehabilitation vs surgical stabilisation	QALY	£1410	- £3304	- 0.068	48,588 £ per QALY (CE threshold 20,000-30,000£ per QALY)
Smeets 2009 [21] Netherland	Combined treatment vs active physical treatment	RMDS	Not reported	- €456	- 1.23	371 € per one point reduction in RMDS
		QALY			0.014	35,060 € per QALY
	Combined treatment vs graded activity plus problem solving	RMDS		€4765	- 1.27	(dominated)
		QALY			- 0.045	(dominated)
Lambeek 2010 [20] Netherlands	Integrated care vs usual care	Return to work (days)	£1077	£217 (direct cost)	- 68	-3^a^ £ per 1 day earlier return to work
		QALY		- £5310 (total cost)	0.09	(dominant)
Johnsen 2014 [18] Norway	Multidisciplinary rehabilitation vs total disc replacement	QALY using EQ-5D	€5977	- €13,506	- 0.34	39748^a^ € per QALY
		QALY using SF-6D			0.11	128,328 € per QALY (CE threshold €74,600 per QALY)

^a^Cost effective, *NoK* Norwegian Krone, *QALY* quality adjusted life years, *USD* United States Dollar

## Discussion

To our knowledge, this is the first systematic review that summarises the current evidence regarding the cost-effectiveness of PMS in CLBP.

This systematic review does not allow conclusive statements about cost effectiveness of PMS to be made for a number of reasons. Firstly, the complex and diverse nature of the interventions, carried out in a range of settings meant that the intervention itself was not a constant. Secondly, the comparators among the included studies varied considerably, affecting realtive costs and effects. Thirdly, patient cohorts in each study were not necessarily comparable across interventions, and, thus, the response to the PMS would also be different. The inclusion criteria in some studies was limited to working adults, which might limit the generalisability of these services to the general population, which include homeworkers, the unemployed and older adults. A trade-off between increasing the generalisibility of studies while limiting the heterogenity by focusing on a specific subset of people with CLBP need to be considered. This could be achieved by using pragmatic RCTs.

In economic evaluation, generalizability is a major issue that need to considered [36]. Several factors may attribute to this limitation including clinical practice patterns, unit costs and healthcare resources use which can be highly varible across different countries and practice settings.

These challenges are augmented by the heterogeneity of back pain, which is as a result of its complex underlying aetiology and clinical course [37] and psychosocial factors that might influence the progression of the condition.

In this review, three studies out of the five have a high risk of bias, due to limitations in RCT methodological design including clear adminstration of co-interventions and variable compliance among groups. Trials of back pain interventions often seem to have methodological limitations leading to a high risk of bias [38]. A Cochrane review of the effectiveness of PMS in CLBP including 41 studies, of which 28 studies were considered to have a high risk of bias [11].

In this review, all economic studies were conducted alongside RCTs and the follow-up period ranged from one to 2 years. Although this length of follow-up gives some idea of the downstream medical cost and outcomes associated with long-term treatment, ideally longer follow-up (lifetime horizon) is needed. According to the MRC recommendations for complex intervention evaluations [39], a lifetime horizon is needed to demonstrate the sustainability of short term changes in outcomes.

Several shortcomings were found in estimating the cost of the PMS. Using a top-down approach gives the average cost per patient; however, the method is not useful in estimating the cost of people who consume healthcare resources more or less than average patients do, such as people with mild or severe conditions. Unit costs were either poorly reported or not reported at all. In addition methods for assessing indirect costs might be inaccurate as presenteeism doesn’t take into account the employee productivity [30].

Apart from methodological limitations in the economic evaluation study design, terminology can be a problem when conducting systematic reviews of complex interventions [40]. Service terminology is highly variable across different settings, countries and clinical contexts and this factor might increase the difficulty in searching and identifying relevant publications. Researchers who are interested in conducting systematic reviews in complex interventions need to select either a search strategy that gathers a wide range of heterogeneous studies to inform the literature about factors that influence this heterogeneity and, thus, address these limitations for future research, or use strict search criteria that define the intervention clearly, which can result in a robust conclusion. However, conducting such a review might be difficult when researchers use different terminology. In addition, narrowing the research might result in a small number of studies, given that the type and intensity of treatments provided within PMS are highly variable. The chance of finding studies that have an identical service, in terms of staff, disciplines involved, duration and intensity, is unlikely.

In this systematic review, a broad search criterion was chosen to conduct the systematic review because the main objective was to investigate the cost effectiveness of the PMS regardless of the type or the intensity of the treatments provided. Although it is logical to compare interventions that have the same components to assess cost effectiveness, it is rare to find studies comparing a matching intervention.

Given these challenges, inconclusive results about the cost effectiveness of these services arise from the variability and heterogeneity of the services and the condition respectively, which makes comparison between studies difficult. The other issue is the poor quality of RCTs, which are the source of “effectiveness data” in economic evaluation, leading to weak cost effectiveness results. Finally, the limitations in estimating the cost of services and the short follow-up period affect the results of the economic evaluations.

The economic evaluations identified in this review were alongside RCTs with short follow-up periods, which is insufficient to assess the long term benefit of the service. Decision analysis models, which usually have lifetime cost effectiveness data, were lacking.

## Conclusion

PMS might be cost effective in the management of CLBP. However, this systematic review highlights the discrepancies in the evidence supporting PMS for people with CLBP due to the variability of the setting, interventions, comparators and outcomes. Therefore, more site specfic and better studies are needed. Research directions should focus on optimising the methods of assessing healthcare resource use and cost in order to improve the analysis in the future. In addition, well conducted RCTs with low risk of bias are needed. Finally,assessing the long terms benefits of these services by conducting pragmatic RCTs combined with model-based economic evaluations is warrented.

## Supplementary information

**Additional file 1.**

**Additional file 2.**

## Data Availability

All data generated or analysed during this study are included in this published article and its supplementary information files”.

## Abbreviations

BPI: Brief Pain Inventory
CBRG: Cochrane Back Review Group
CBT: Cognitive behavioural therapy
CEAC: Cost effectiveness acceptability curve
CHEERS: Consolidated Health Economic Evaluation Reporting Standards
CLBP: Chronic low back pain
EQ 5D: EuroQol 5 dimensions
GP: General practitioner/practice
HTA: Health technology assessment
IASP: International Association for the Study of Pain
ICER: Incremental cost effectiveness ratio
IMMPACT: Initiative on Methods, Measurement and Pain Assessment in Clinical Trials
INMB: Incremental net monetary benefit
ISPOR: International Society for Pharmacoeconomics and Outcomes Research
NICE: National Institute for Health and Care Excellence
ODI: Oswestry Disability Index
PMS: Pain management service
QALY: Quality adjusted life years
RCT: Randomised controlled trials
RMDQ: Roland Morris Disability Questionnaire
SC: Standard care
SMD: Standardised Mean Difference
