# Supplementary material for: Cost-effectiveness of pain management services for chronic low back pain: a systematic review of published studies
Source: BMC Health Serv Res. 2020 Mar 12;20:194. doi: 10.1186/s12913-020-5013-1 (PMC7069015; doi:10.1186/s12913-020-5013-1)
Supplement: Supplementary file 1 — Additional file 1. [file 12913_2020_5013_MOESM1_ESM.docx]

**Additional file 1**

**Search strategy**

**Back pain**

coccyx.ti,ab.

coccydynia.ti,ab.

back disorder$.ti,ab.

dorsalgia.ti,ab.

exp Back Pain/

backache.ti,ab.

exp Low Back Pain/

(lumbar adj pain).ti,ab.

back pain.ti,ab.

1 or 2 or 3 or 4 or 5 or 6 or 7 or 8 or 9

**Multidicsplinary pain clinics**

multidisciplinar$.tw.

interdisciplinar$.tw.

multiprofessional$.tw.

multimodal$.tw.

patient care team.sh.

exp patient care management/

pain clinics.sh.

(pain clinic$ or pain center$ or pain service$ or pain reliefe unit$ or pain center$).tw.

rehabilitation, vocational.sh.

rehabilitation centers.sh.

rehabilitation clinic$.tw.

rehabilitation.ti,ab.

11 or 12 or 13 or 14 or 15 or 16 or 17 or 18 or 19 or 20 or 21 or 22

**Economic studies**

Economics/

"costs and cost analysis"/

Cost allocation/

Cost-benefit analysis/

Cost control/

Cost savings/

Cost of illness/

Cost sharing/

"deductibles and coinsurance"/

Medical savings accounts/

Health care costs/

Direct service costs/

Drug costs/

Employer health costs/

Hospital costs/

Health expenditures/

Capital expenditures/

Value of life/

exp economics, hospital/

exp economics, medical/

Economics, nursing/

Economics, pharmaceutical/

exp "fees and charges"/

exp budgets/

(low adj cost).mp.

(high adj cost).mp.

(health?care adj cost$).mp.

(fiscal or funding or financial or finance).tw.

(cost adj estimate$).mp.

(cost adj variable).mp.

(unit adj cost$).mp.

(economic$ or pharmacoeconomic$ or price$ or pricing).tw.

Cost effectiveness analysis/

Cost utility analysis/

**Combining back pain, multidisciplinary pain clinic and economic studies**

24 or 25 or 26 or 27 or 28 or 29 or 30 or 31 or 32 or 33 or 34 or 35 or 36 or 37 or 38 or 39 or 40 or 41 or 42 or 43 or 44 or 45 or 46 or 47 or 48 or 49 or 50 or 51 or 52 or 53 or 54 or 55 or 56 or 57

10 and 23 and 58

**Deduplication and limitation**

remove duplicates from 59

limit 60 to humans

limit 61 to English language
