# Supplementary material for: Cost-effectiveness of pain management services for chronic low back pain: a systematic review of published studies
Source: BMC Health Serv Res. 2020 Mar 12;20:194. doi: 10.1186/s12913-020-5013-1 (PMC7069015; doi:10.1186/s12913-020-5013-1)
Supplement: Supplementary file 2 — Additional file 2. [file 12913_2020_5013_MOESM2_ESM.docx]

**Additional file 2**

**List of excluded studies and reasons for exclusion**

| **Study** | **Reason for exclusion** |
| --- | --- |
| Whitehurst 2007 (1) | Acute back pain |
| Strong 2006 (2) | Single intervention |
| Fritzell 2004 (3) | Not Multidisciplinary intervention |
| Jensen 2013 (4) | Mixed conditions |
| Rogerson 2009(5) | Acute LBP |
| Jensen 2009 (6) | Mixed conditions |
| Sogaard 2008 (7) | Post-surgery |
| Goossens 1998 (8) | Single intervention |
| Hill 1996 (9) | Partial economic evaluation |
| Kominski 2005 (10) | Partial economic evaluation |
| Loisel 2002 (11) | Sub-acute pain |
| Berenguera 2011, lambeek 2007, van der Roer 2004 (12-14) | Protocols |
| Weh 2011(15) | Abstract |
| Henchoz 2010 (16) | Different intervention |
| Uhlig 2003 (17) | Review |

**References**

1. Whitehurst DG, Lewis M, Yao GL, Bryan S, Raftery JP, Mullis R, et al. A brief pain management program compared with physical therapy for low back pain: results from an economic analysis alongside a randomized clinical trial. Arthritis and rheumatism. 2007;57(3):466-73.

2. Strong LL, Von Korff M, Saunders K, Moore JE. Cost-effectiveness of two self-care interventions to reduce disability associated with back pain. Spine (Phila Pa 1976). 2006;31(15):1639-45.

3. Fritzell P, Hagg O, Jonsson D, Nordwall A. Cost-effectiveness of lumbar fusion and nonsurgical treatment for chronic low back pain in the Swedish Lumbar Spine Study: a multicenter, randomized, controlled trial from the Swedish Lumbar Spine Study Group. Spine (Phila Pa 1976). 2004;29(4):421-34; discussion Z3.

4. Jensen C, Nielsen CV, Jensen OK, Petersen KD. Cost-effectiveness and cost-benefit analyses of a multidisciplinary intervention compared with a brief intervention to facilitate return to work in sick-listed patients with low back pain. Spine (Phila Pa 1976). 2013;38(13):1059-67.

5. Rogerson MD, Gatchel RJ, Bierner SM. A cost utility analysis of interdisciplinary early intervention versus treatment as usual for high-risk acute low back pain patients. Pain practice : the official journal of World Institute of Pain. 2010;10(5):382-95.

6. Jensen IB, Busch H, Bodin L, Hagberg J, Nygren A, Bergstrom G. Cost effectiveness of two rehabilitation programmes for neck and back pain patients: A seven year follow-up. Pain. 2009;142(3):202-8.

7. Sogaard R, Bunger CE, Laurberg I, Christensen FB. Cost-effectiveness evaluation of an RCT in rehabilitation after lumbar spinal fusion: a low-cost, behavioural approach is cost-effective over individual exercise therapy. European spine journal : official publication of the European Spine Society, the European Spinal Deformity Society, and the European Section of the Cervical Spine Research Society. 2008;17(2):262-71.

8. Goossens ME, Rutten-Van Molken MP, Kole-Snijders AM, Vlaeyen JW, Van Breukelen G, Leidl R. Health economic assessment of behavioural rehabilitation in chronic low back pain: a randomised clinical trial. Health Econ. 1998;7(1):39-51.

9. Hill PA, Hardy PAJ. The cost-effectiveness of a multidisciplinary pain management programme in a district general hospital: Pain Clinic. 9 (2) (pp 181-188), 1996. Date of Publication: 1996.; 1996.

10. Kominski GF, Heslin KC, Morgenstern H, Hurwitz EL, Harber PI. Economic evaluation of four treatments for low-back pain: results from a randomized controlled trial. Medical care. 2005;43(5):428-35.

11. Loisel P, Lemaire J, Poitras S, Durand MJ, Champagne F, Stock S, et al. Cost-benefit and cost-effectiveness analysis of a disability prevention model for back pain management: a six year follow up study. Occupational and environmental medicine. 2002;59(12):807-15.

12. Lambeek LC, Anema JR, van Royen BJ, Buijs PC, Wuisman PI, van Tulder MW, et al. Multidisciplinary outpatient care program for patients with chronic low back pain: design of a randomized controlled trial and cost-effectiveness study [ISRCTN28478651]. BMC public health. 2007;7:254.

13. van der Roer N, van Tulder MW, Barendse JM, van Mechelen W, Franken WK, Ooms AC, et al. Cost-effectiveness of an intensive group training protocol compared to physiotherapy guideline care for sub-acute and chronic low back pain: design of a randomised controlled trial with an economic evaluation. [ISRCTN45641649]. BMC Musculoskelet Disord. 2004;5:45.

14. Berenguera A, Pujol-Ribera E, Rodriguez-Blanco T, Violan C, Casajuana M, de Kort N, et al. Study protocol of cost-effectiveness and cost-utility of a biopsychosocial multidisciplinary intervention in the evolution of non-specific sub-acute low back pain in the working population: cluster randomised trial. BMC musculoskeletal disorders. 2011;12:194.

15. Weh L, Marnitz U, Bromme J. Interdisciplinary multimodal therapie versus conventional treatment of chronic back pain: A cost analysing matched pairs-study. European Spine Journal. 2011;20 (11):1981.

16. Henchoz Y, Pinget C, Wasserfallen JB, Paillex R, de Goumoens P, Norberg M, et al. Cost-utility analysis of a three-month exercise programme vs usual care following multidisciplinary rehabilitation for chronic low back pain. Journal of rehabilitation medicine. 2010;42(9):846-52.

17. Uhlig T, Finset A, Kvien TK. Effectiveness and cost-effectiveness of comprehensive rehabilitation programs. Current opinion in rheumatology. 2003;15(2):134-40.
